# Zinc Protects Oxidative Stress-Induced RPE Death by Reducing Mitochondrial Damage and Preventing Lysosome Rupture

**DOI:** 10.1155/2017/6926485

**Published:** 2017-11-14

**Authors:** Dinusha Rajapakse, Tim Curtis, Mei Chen, Heping Xu

**Affiliations:** Centre for Experimental Medicine, School of Medicine, Dentistry & Biomedical Sciences, Queen's University Belfast, 97 Lisburn Road, Belfast BT9 7BL, UK

## Abstract

Zinc deficiency is known to increase the risk of the development of age-related macular degeneration (AMD), although the underlying mechanism remains poorly defined. In this study, we investigated the effect of zinc on retinal pigment epithelium (RPE) survival and function under oxidative conditions. Zinc level was 5.4 *μ*M in normal culture conditions (DMEM/F12 with 10% FCS) and 1.5 *μ*M in serum-free medium (DMEM/F12). Under serum-free culture conditions, the treatment of RPE cells with oxidized photoreceptor outer segment (oxPOS) significantly increased intracellular ROS production, reduced ATP production, and promoted RPE death compared to oxPOS-treated RPE under normal culture condition. Serum deprivation also reduced RPE phagocytosis of oxPOS and exacerbated oxidative insult-induced cathepsin B release from lysosome, an indicator of lysosome rupture. The addition of zinc in the serum-free culture system dose dependently reduced ROS production, recovered ATP production, and reduced oxidative stress- (oxPOS- or 4-HNE) induced cell death. Zinc supplementation also reduced oxidative stress-mediated cathepsin B release in RPE cells. Our results suggest that zinc deficiency sensitizes RPE cells to oxidative damage, and zinc supplementation protects RPE cells from oxidative stress-induced death by improving mitochondrial function and preventing lysosome rupture.

## 1. Introduction

Zinc is the second most prominent trace element in the human body with the majority stored in skeletal muscle and the rest distributed between the bone, liver, skin, and other tissues [1]. In the circulation, around 80% of zinc is loosely bound to albumin. When circulating levels drop, gastrointestinal absorption of dietary zinc increases to maintain systemic homeostasis. In cells, zinc is distributed within the cytoplasm, nucleus, and membrane. The majority of cellular zinc is bound with proteins and sequestered into organelles resulting in low levels of labile or free zinc. Labile zinc, defined as zinc not bound to proteins, varies among different cellular compartments with 0.14 pM in the mitochondria, 0.9 pM in the endoplasmic reticulum (ER), and 0.2 pM in the Golgi [1]. Zinc plays an important structural role in proteins by the formation of the zinc finger motif [2]. It can also be released extracellularly and functions as a signaling molecule by binding to cell surface receptors to trigger second messenger responses [3]. In addition, intracellular zinc can modulate cell signaling by targeting various kinase-dependent pathways, including the MAPK and PKC pathways [4].

Zinc is the most abundant trace metal present in the retina [5] and is found mainly in the retinal pigment epithelium (RPE) and photoreceptor layers as well as in organelles including Golgi apparatus, melanosomes, and lysosomes [6–8]. Apart from its role as a cofactor for several antioxidant enzymes [9], zinc is also involved in the visual cycle through regulation of retinol dehydrogenase and rhodopsin regeneration [10] and can metabolize ingested photoreceptor outer segments (POS) in RPE cells [11]. In normal conditions, cellular zinc concentration is tightly regulated within a concentration range that is nontoxic. Cells maintain zinc homeostasis by regulating zinc-sequestering proteins such as metallothioneins and/or altering the expression of zinc transporter proteins which transport zinc between extracellular and intracellular regions and within intracellular compartments [12]. Zinc deficiency occurs in a number of pathologies, including epilepsy, Alzheimer's disease, and age-related macular degeneration (AMD) [13]. This is thought to arise due to the failure of regulating proteins that are important for zinc homeostasis or underlying dietary zinc deficiency [13]. Inadequate levels of serum zinc have been frequently observed in patients with age-related disease [14]. With regard to AMD, ageing RPE cells display abundant lipofuscin accumulation, while the content of zinc-containing melanosomes decreases [15]. Melanosomes are the primary zinc reservoir in pigmented tissues, and it is reported that zinc deficit is most common in elderly populations who are prone to AMD pathogenesis [16]. Erie et al. [17] demonstrated a 24% reduction in the zinc level of the RPE-choroid complex in donors with AMD compared with non-AMD donors, suggesting that zinc homeostasis may be implicated in AMD and retinal health. Indeed, zinc supplementation in AMD patients has demonstrated a beneficial effect in terms of AMD progression [18]. At a mechanistic level, zinc deficiency has been shown to promote lipid peroxidation which subsequently impairs RPE cell phagocytic and lysosomal activity [19]. Such changes may be linked to the accumulation of lipofuscin in RPE cells seen during aging. Zinc-deficient animals, for example, exhibit increased formation of lipofuscin through a pathway involving elevated oxidative stress and incomplete digestion of POS in the lysosomes of the RPE [16]. Failure to control zinc homeostasis has also been suggested to contribute to the aggregation of immunoproteins such as complement factor H (CFH) found in sub-RPE deposits in patients with AMD [20]. The mechanism by which zinc deficiency may contribute to the development of AMD, however, has yet to be fully elucidated.

Although zinc deficiency is a potential contributing factor to the progression of AMD, excessive intracellular zinc can also result in RPE cytotoxicity [21], for example, Wood and Osborne showed that 18 *μ*M zinc decreased human RPE viability by 50%, which was reversed following supplementation with metabolic substrates and antioxidants. These findings are of interest because the in vivo level of zinc in RPE is usually high under normal conditions, and the cells do not appear to undergo significant oxidative damage. Tate et al. [22] showed that RPE cells cultured under low-zinc conditions are more susceptible to oxidative insults and that SOD activity increased in low-zinc medium in response to oxidative stressors. Other studies have also shown that zinc treatment can limit RPE cell oxidative stress induced by cadmium, a metal ion in cigarette smoke [23, 24]. Clearly, the role of zinc in RPE biology and function warrants further exploration.

We have shown previously that continuous exposure of POS and oxidized POS (oxPOS) results in a buildup of ROS in RPE cells, yet cells remained healthy and functional [25], suggesting that there are components within RPE enabling homeostasis despite this insult. The aim of this study was to determine the role of zinc in maintaining RPE cell homeostasis in cultures when exposed to low levels of oxidative insults similar to those seen with aging and age-related diseases (e.g., oxPOS or a low concentration of 4-HNE).

## 2. Materials and Methods

### 2.1. In Vitro RPE Cell Culture

ARPE19 cells were purchased from ATCC (CRL-2302, Middlesex, UK). RPE cells were cultured in complete Dulbecco's Modified Eagle Medium/F12 (DMEM/F12) medium containing 10% fetal calf serum (FCS) (Gibco Life Technology, UK) and 100 *μ*g/mL Primocin (Invitrogen, San Diego, California, USA). Cells were maintained in an incubator at 37°C with 5% CO_2_ using T25 and T75 tissue culture flasks or tissue culture plates (Nunc™ Surface, Leicestershire, UK). The phenotype of RPE cells was confirmed by pancytokeratin staining. Passages 5–10 cells were used in the study.

### 2.2. Preparation of Photoreceptor Outer Segments

POS were isolated from bovine eyes using the method described previously by Chen et al. [26]. In brief, 20 bovine retinas were placed in 20 ml homogenizing solution made up of 20% *w*/*v* sucrose, 20 mM Tris-acetate pH 7.2, 2 mM MgCl2, 10 mM glucose, and 5 mM taurine. The suspension was shaken for 1 min and filtered through a 100 mm cell strainer (BD, Oxford, UK) to remove tissue debris. The suspension was then carefully layered on 25–60% *w*/*v* continuous sucrose gradients containing 20 mM Tris-acetate pH 7.2, 10 mM glucose, and 5 mM taurine and centrifuged at 25,000 rpm for 45 min at 4°C. The pink band containing POS was collected and washed with storage buffer comprising of 10 mM sodium phosphate pH 7.2, 0.1 M NaCl, and 2.5% sucrose. Isolated POS aliquots were stored at −80°C at a concentration of 10^8^ POS/ml.

### 2.3. Oxidized POS (oxPOS) Generation

The isolated POS aliquots were transferred to 9 cm petri dishes and exposed to 302 nm ultraviolet light (Ultraviolet Products, Cambridge, UK) in a laminar airflow box for 12 h to produce oxPOS. Samples were then collected and washed with distilled water. The irradiated POS were pelleted by centrifugation at 12,000*g* for 20 min and resuspended in storage buffer, and lipid oxidation was confirmed by the thiobarbituric acid reactive substance assay kit (Alexis; Axxora Ltd, Nottingham, UK). When treating RPE cells with oxPOS, a ratio of 1 : 10 RPE and oxPOS was used.

### 2.4. Zinc Depletion by Serum Starvation

Total zinc level in the complete medium (10% serum) is 5.4 *μ*M [27]. To deplete total zinc in culture to 1.5 *μ*M (Ref: http://www.thermofisher.com/us/en/home/technical-resources/media-formulation.55.html), culture media was removed and the cells were washed once with serum-free medium (SFM) before reincubating in SFM and DMEM/F12 with 100 *μ*g/mL Primocin for 48 h. For supplementation with zinc, ZnCl_2_ was prepared in a 15 *μ*M concentration in SFM and filtered through a 0.22 *μ*g filter and added to cells after 24 h.

### 2.5. Immunofluorescence Staining

ARPE19 cells were cultured on cover slips under different treatment conditions, including oxPOS and zinc depleted and zinc supplemented for 48 h. Cells were washed with cold PBS and fixed with 1% paraformaldehyde (PFA) for 10 min, followed by permeabilization with 0.1% Triton-X for 5 min. The samples were then blocked with 5% BSA for 30 min at room temperature and incubated with anti-human cathepsin B (1 : 100, Life Technologies, UK) antibody for 2 h. After washing with PBS, samples were incubated with anti-mouse IgG 488 secondary antibody at a dilution of 1 : 100 with PBS and nuclei stained with DAPI at a dilution of 1 : 500 (Life Technologies, UK) in the dark for 1 h. Following thorough washing, coverslips were mounted with mounting medium (Vector Laboratories, Peterborough, UK) and examined by confocal microscopy (Eclipse TE2000-U, Nikon, Surrey, UK) at 40x magnification and numerical aperture at 0.65.

Dye-conjugated molecular probes used for staining intracellular organelles or other molecules used in this study are listed in Table 1. The dyes were added to live cells overnight in the dark at 37°C according to the manufacturers' instructions. After washing, fresh media was added and the samples were imaged by confocal microscopy.

### 2.6. Detection of Reactive Oxygen Species (ROS) Using CellROX Green

ARPE19 cells cultured on coverslips or 96-F fluorescence microplates (black) with/without oxPOS, under control and zinc depleted and zinc-supplemented conditions, were treated with 5 *μ*M per well CellROX green (Life Technologies) for 30 min at 37°C.

The fluorescence intensity of CellROX green in cells was quantified using a fluorescent plate reader with an excitation filter of 485 nm and an emission filter of 520 nm. The background fluorescence intensity/autofluorescence was subtracted from the CellROX green intensity of cells for data analysis. For confocal microscopy, cells were further stained with MitoTracker Red (Life Technologies) and Hoechst 33342 (Thermo Fisher Scientific Loughborough, UK) for a further 30 min.

### 2.7. In Situ Cell Death Detection Terminal Deoxynucleotidyl Transferase (TdT) dUTP Nick-End Labeling (TUNEL) Assay

RPE cell death was detected using a *TUNEL* assay kit (Sigma-Aldrich, UK) according to the manufacturer's instructions. In brief, RPE cells plated for experiments with oxPOS or 4-HNE were washed with cold PBS and then fixed with 4% paraformaldehyde (PFA) for 1 h at 15–25°C before washing with PBS. The samples were then permeabilized with 0.1% Triton-X for 2 min on ice (2–8°C). As a negative control, one well contained labelling solution only. As a positive control, 3 U/ml recombinant DNASE1 was added to one group of cells to induce DNA strand breaks prior to labelling. The complete TUNEL reaction mixture was added to all experimental wells and incubated at 37°C for 1 h. The cells were washed with PBS before imaging under a confocal microscope. A series of 4-HNE concentrations were tested to choose a concentration that induced 50% or less cell death in 48 h treatment (data not shown). Therefore, apart from oxPOS, 5 *μ*M 4-HNE for 24 h was used as an alternative oxidative insult to induce RPE cell death.

### 2.8. ToxGlo Assay to Measure Mitochondria ATP Production

RPE cells were plated on 96-well plates with clear or solid bottoms ensuring even dispersion and incubated at 37°C. Each treatment group (control untreated RPE cells, oxPOS treated, 1.5 *μ*M zinc, 1.5 *μ*M zinc + oxPOS treated, 15 *μ*M zinc supplemented + oxPOS treated, and 15 *μ*M zinc supplemented) was plated in 6 experimental wells. Five hours later and following cell attachment, zinc depleted media and oxPOS treatments were added to experiment wells. At 24 h, 15 *μ*M zinc-supplemented media was added to the relevant experiment well for another 24 h. The cells were then washed with PBS, followed by incubation with cytotoxicity reagent (provided with assay kit) for 30 min. Fluorescence was measured at 485 nm/520–530 nm using a microplate reader (BMG Labtech, Germany). The assay plate was left to equilibrate at room temperature for 5–10 min, and 100 *μ*l of ATP detection reagent was added to each well. The plate was mixed by orbital shaking 500–700 rpm for 1–5 min. Luminescence was measured using a microplate reader (BMG Labtech, Germany).

### 2.9. Western Blot

ARPE19 cells were washed with 1X phosphate-buffered saline and lysed in RIPA buffer with protease inhibitors (Sigma-Aldrich, UK). The protein concentrations were measured using a BCA protein assay kit (PIERCE, Cramlington, UK). 20 *μ*g of the total protein was loaded onto 10% SDS-PAGE gel. Gels were run at 80 V for 30 min followed by 150 V for 60 min. Proteins were transferred to the Immobilon-FL polyvinylidene difluoride (PVDF) membrane (Millipore, Watford, UK) at 350 mA for 50 min. Blots were blocked with 5% bovine serum albumin (BSA) in tris-buffered saline with Tween-20 (TBS/T) for 1 h at room temperature then rinsed once in TBS/T. Next, the blots were incubated with rabbit polyclonal anti-Beclin-1 (Abcam) and *β*-actin (Santa Cruz) primary antibodies diluted 1 : 1000 with TBS/T overnight at 4°C. After thorough washes, the membranes were incubated with conjugated secondary antibodies at 1 : 1000 dilutions for 2 h in the dark at room temperature. The secondary antibodies used were goat anti-rabbit IRDye 680 (1 : 5000) and goat anti-mouse IRDye 800 (1 : 5000); (LI-COR; Lincoln).

The membrane was then washed in TBS/T 3 times before scanning using Odyssey infrared imaging system (Li-COR Biotechnology, Cambridge, UK). Quantitative Western blotting was performed using ImageJ software (version 1.45). The blots shown are representative of at least three biologic repeats of each experiment. The *β*-actin level was used to normalize the signal.

### 2.10. Data and Statistical Analysis

All experiments were performed in biological triplicate for statistical analysis. The data are expressed as mean ± SEM with *P* < 0.05 deemed statistically significant. Differences between groups were assessed using either an independent *t*-test or one-way analysis of variance with Dunnett's or Tukey's post hoc tests.

## 3. Results

### 3.1. Serum Starvation in Culture Reduces Intracellular Labile Zinc in ARPE19 Cells

The zinc concentration in control culture conditions with DMEM/F12+ 10% FCS is maintained at 5.4 *μ*M, and in serum-free DMEM/F12 medium is 1.5 *μ*M. In serum, 80% of zinc is loosely bound to albumin and the remaining 20% are present as labile zinc [1], therefore when culture media is supplemented with 15 *μ*M zinc, there is approximately twofold increase in zinc bioavailability.

**Figure 1:**
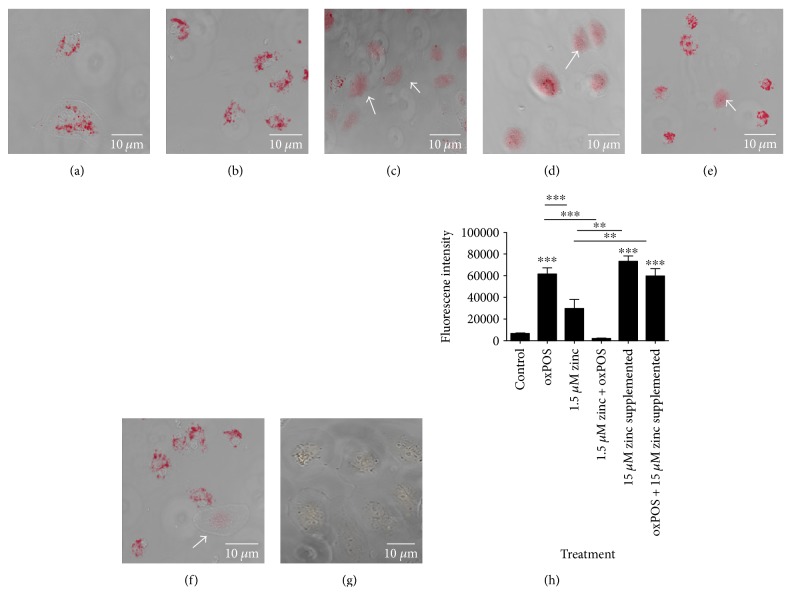
Confocal images of intracellular zinc using FluoZin-3 AM live cell staining. RPE cells treated under different conditions were incubated with 5 *μ*M FluoZin-3 AM for 30 min at 37°C in the dark. The cells were then washed and further incubated for 30 min before imaging using a confocal microscope. (a) Control RPE cells cultured in 10% serum. (b) oxPOS-treated RPE cells in 10% serum. (c) RPE cells cultured in serum-free 1.5 *μ*M zinc. (d) RPE cells cultured in serum-free 1.5 *μ*M zinc + oxPOS. (e) RPE cells cultured in serum-free 15 *μ*M zinc supplement + oxPOS. (f) RPE cells cultured in serum-free 15 *μ*M zinc supplement. (g) Control RPE cells showing autofluorescence of oxPOS. Arrows show cells with decreased intracellular zinc staining. (h) Histogram showing fluorescence intensity of FluoZin-3 AM in different treatment groups of cells, ^∗∗^*P* < 0.01 and ^∗∗∗^*P* < 0.001. Data were analyzed by one-way ANOVA followed by Tukey's multiple comparison test. *N* = 50.

Under normal culture conditions, RPE cells contain a significant amount of intracellular labile zinc and levels were further increased following oxPOS incubation (Figures 1(a) and 1(b)). After 48 h culture in serum-free DMEM/F12 condition, the intracellular levels of zinc in RPE cells decreased significantly compared to that of cells (DMEM/F12+ 10%FCS) with/without oxPOS (Figures 1(a), 1(d), and 1(h)). However, after supplementation with 15 *μ*M zinc for 24 h, the levels of intracellular zinc improved significantly (Figures 1(e), 1(f), and 1(h)).  Figure 1(g) shows control RPE cells without FluoZin-3 AM showing autofluorescence of oxPOS which confirms there is minimal background fluorescence. Our results therefore substantiated the serum deprivation protocol resulted in intracellular zinc depletion, and the decrease in intracellular zinc is due to the reduced zinc in extracellular regions.

### 3.2. Zinc Depletion Induces Cellular Oxidative Stress

**Figure 2:**
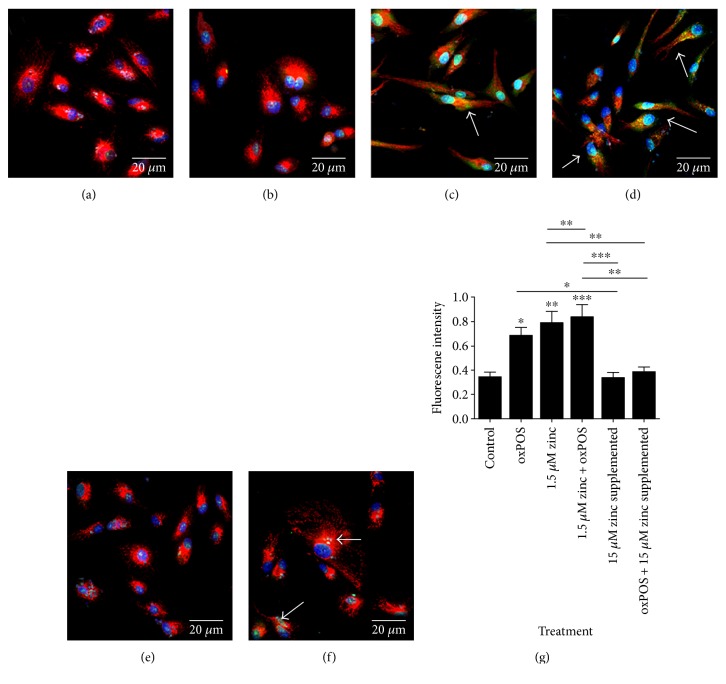
CellROX staining of oxidant generation in RPE following oxidative stress and zinc depletion. RPE cells under different treatment conditions were incubated with 5 *μ*M per well concentration of CellROX for 30 min at 37°C, followed by MitoTracker and Hoechst 33,342. Cells were washed and fresh medium was added to the wells and imaged by confocal microscopy (a–f) or analyzed by a fluorescent plate reader (g). (a) Control RPE cells in 10% serum. (b) oxPOS-treated RPE cells in 10% serum. (c) Serum-free RPE cells in 1.5 *μ*M zinc. (d) Serum-free RPE cells in 1.5 *μ*M zinc + oxPOS. (e) 15 *μ*M zinc-supplemented RPE cells. (f) oxPOS + 15 *μ*M zinc-supplemented RPE cells. Red: MitoTracker; blue: nuclei Hoechst 33,342; and green: oxidant DNA in CellROX. (g) Histogram showing fluorescence intensity of CellROX green in different treatment groups of cells analyzed by a fluorescent plate reader. Mean ± SEM is plotted for 6 replicates for each condition. ^∗^*P* < 0.05, ^∗∗^*P* < 0.01, and ^∗∗∗^*P* < 0.001. Data analyzed by one-way ANOVA followed by Tukey's multiple comparison test.

To determine the effects of zinc depletion on the buildup of ROS in RPE cells after exposure to oxPOS, intracellular ROS was measured using CellROX green. oxPOS treatment alone significantly increased oxidant generation in RPE cells (Figures 2(a), 2(b), and 2(g)); however, there was a greater increase in CellROX staining in the cells cultured in DMEM/F12 serum-free medium (1.5 *μ*M Zinc, Figures 2(c) and 2(g)). The combination of low zinc + oxPOS treatment further increased CellROX staining (Figures 2(c), 2(d), and 2(g)). After supplementation with 15 *μ*M zinc, there was a significant decrease in the intensity of CellROX staining in both groups that were cultured in serum-free condition (i.e., low zinc and low zinc + oxPOS) (Figures 2(e), 2(f), and 2(g)). The results suggest that zinc has an antioxidant role in RPE cells.

### 3.3. Zinc Depletion Induces Mitochondria Morphology Disorganization and Functional Changes

**Figure 3:**
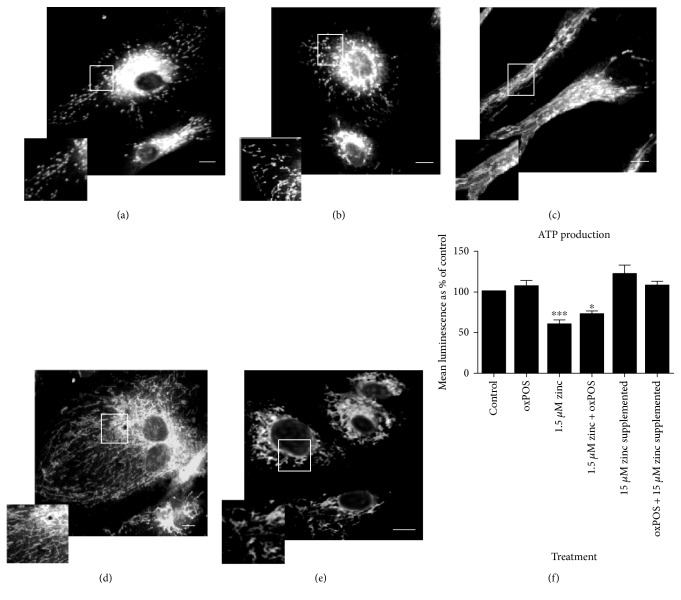
Changes in mitochondria morphology staining and function in a stressed environment. RPE was subjected to treatments with 1.5 *μ*M zinc, 1.5 *μ*M zinc + oxPOS treated, oxPOS treated + 15 *μ*M zinc supplemented, and oxPOS-treated untreated control for 48 h. MitoTracker was added for 30 min at 37°C before imaging the cells living under the confocal microscope at 60x magnification or samples were processed for Mitochondrial ToxGlo assay to measure ATP production. (a) Control-untreated RPE cells. (b) oxPOS-treated RPE cells. (c) 1.5 *μ*M zinc RPE cells. (d) 1.5 *μ*M zinc + oxPOS-treated RPE cells. (e) 15 *μ*M zinc-supplemented RPE cells. (f) Quantitative data of mitochondria ATP production in different treatment groups, ^∗^*P* < 0.05 and ^∗∗∗^*P* < 0.001, compared to untreated control with data analyzed using a one-way ANOVA followed by Dunnett's multiple comparison test. *N* = 5.

RPE cells cultured under control complete medium with/without oxPOS demonstrate normal mitochondrial morphology (Figures 3(a) and 3(b)), whereas cells cultured under serum-free conditions (1.5 *μ*M zinc and 1.5 *μ*M zinc + oxPOS) exhibit fragmented to elongated string-like mitochondrial morphology (Figures 3(c) and 3(d)). This change in morphology is known to be related to mitochondrial fission and fusion, which often occur when cells are under metabolic or environmental stress conditions [27]. When cells were treated with 15 *μ*M zinc supplementation for 24 h, the changes observed in mitochondrial morphology was reversed (Figure 3(e)).

The mitochondrial ATP production was also partially but significantly decreased after serum deprivation (1.5 *μ*M zinc). Interestingly, the addition of oxPOS did not further decrease ATP production. However, when 15 *μ*M zinc was supplemented, ATP production returned to a level comparable to control RPE cells (Figure 3(f)). Our results suggest that zinc plays a role in maintaining mitochondrial function and ATP production.

### 3.4. Zinc Depletion Sensitizes ARPE19 Cells to Additional Oxidative Insults

**Figure 4:**
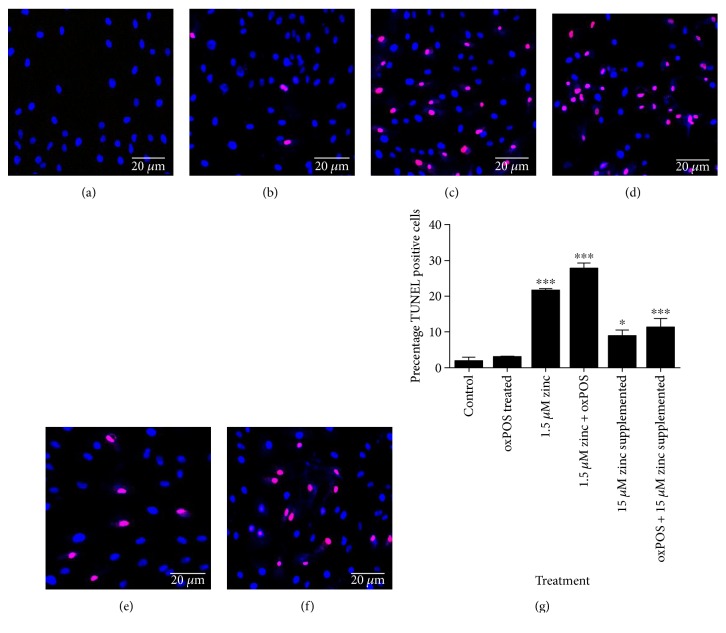
In situ cell death staining to detect RPE cell apoptosis in oxPOS treated and zinc depletion. RPE cells were subjected to treatments with1.5 *μ*M zinc, 1.5 *μ*M zinc + oxPOS treated, oxPOS treated + 15 *μ*M zinc supplemented, and oxPOS-treated and untreated control for 48 h were fixed and processed for TUNEL assay. (a) Control-untreated RPE cells. (b) oxPOS-treated RPE cells. (c) 1.5 *μ*M zinc RPE cells. (d) 1.5 *μ*M zinc + oxPOS-treated RPE cells. (e) 15 *μ*M zinc-supplemented RPE cells. (f) oxPOS + 15 *μ*M zinc-supplemented RPE cells. The cell nucleus is stained with DAPI (blue). Red staining indicates TUNEL positive cells. (g) Histogram showing the percentage of TUNEL positive cells in each treatment groups, ^∗^*P* < 0.05 and ^∗∗∗^*P* < 0.001, compared to each group with the data analyzed by one-way ANOVA followed by Tukey's multiple comparison test. 200 cells were counted for each well. *N* = 3.

Under normal culture conditions, 3% TUNEL^+^ cells were detected (Figures 4(a) and 4(g)). The addition of oxPOS did not significantly increase the percentage of TUNEL^+^ cells after 48 h (Figures 4(b) and 4(g)). When RPE cells were cultured in serum-free conditions (1.5 *μ*M zinc), the percentage of TUNEL^+^ cells were significantly increased (Figures 4(c) and 4(g)). The addition of oxPOS further increased the percentage of TUNEL positive cells (Figures 4(d) and 4(g)). Supplementation of 15 *μ*M zinc for 24 h to serum-free RPE cells significantly reduced the percentage of TUNEL^+^ cells (Figures 4(e) and 4(f)).

**Figure 5:**
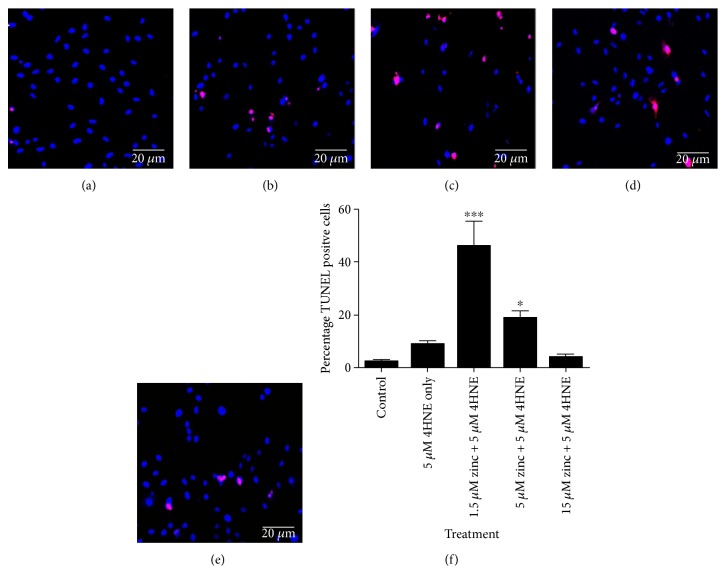
Dose-dependent effect of zinc on 4-HNE-induced RPE cell death. RPE cells subjected to different treatments were fixed and processed for TUNEL assay. (a) Control RPE cells. (b) 5 *μ*M 4-HNE only-treated RPE cells. (c) 1.5 *μ*M zinc + 4-HNE-treated RPE cells. (d) 5 *μ*M zinc + 4-HNE-treated RPE cells. (e) 15 *μ*M zinc + 4-HNE-treated RPE cells. Red nuclei staining indicates TUNEL positive cells. (f) Histogram showing the percentage of TUNEL positive cells in each treatment groups, ^∗^*P* < 0.05 and ^∗∗∗^*P* < 0.001, compared to control with data analyzed by one-way ANOVA followed by Dunnett's multiple comparison test. 100 cells were counted for each well. *N* = 3.

To further investigate the effect of zinc on oxidative stress-induced RPE cell death, ARPE19 cells in normal (DMEM/F12 with 10% FCS) or serum-free (DMEM/F12 only) culture conditions were subjected to 5 *μ*M 4-HNE for 24 h. Under normal culture conditions, there was 3% cell death (Figures 5(a) and 5(f)). 5 *μ*M 4-HNE treatment resulted in 10% cell death (Figures 5(b) and 5(f)), which increased to approximately 45% under serum-free conditions (Figures 5(c) and 5(f)). Zinc supplementation (5 *μ*M and 15 *μ*M zinc) dose dependently suppressed 4-HNE (5 *μ*M)-induced RPE death (Figures 5(d), 5(e), and 5(f)).

Taken together, our results suggest that serum deprivation sensitizes RPE cells to oxidative insult-induced death, and zinc supplementation can protect RPE cells from oxidative stress-induced death under serum-free conditions.

### 3.5. Zinc Depletion Partially Decreases ARPE19 Cell Phagocytosis Function

**Figure 6:**
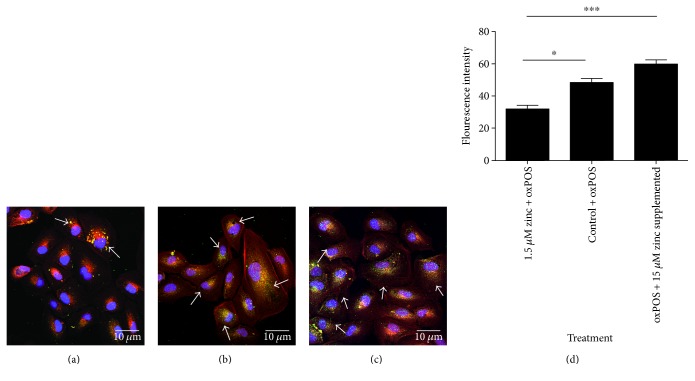
Changes in oxPOS phagocytosis in zinc-depleted RPE. The following zinc was depleted in RPE for 48 h; the cells were exposed to FITC-conjugated oxPOS particles (RPE: oxPOS = 1:10) for 2 h. Cells were then washed, fixed, and processed for imaging under the confocal microscope. The phagocytosis function was quantified by measuring the fluorescent intensity of the phagocytosed particles per cell. (a) 1.5 *μ*M zinc RPE cells + oxPOS-treated. (b) Control RPE cells + oxPOS-treated. (c) oxPOS + 15 *μ*M zinc-supplemented RPE cells. Arrows show phagocytosed oxPOS particles in cells. Cell nuclei are stained with DAPI (blue), actin with phalloidin (red), and oxPOS particles conjugated with FITC (green). (d) Histogram showing fluorescence intensity of the phagocytosed oxPOS particles in the different groups, ^∗^*P* < 0.05 and ^∗∗∗^*P* < 0.001. Data analyzed by one-way ANOVA followed by Tukey's multiple comparison test. 30 cells were counted for each well.

Zinc has been previously been implicated in various RPE functions, and zinc deficiency is associated with disease condition such as AMD [23]. Phagocytosis of photoreceptor outer segments by RPE cells is important and essential to maintain visual function. Under serum-free conditions (1.5 *μ*M zinc), RPE cells phagocytosed significantly fewer oxPOS particles compared to cells in normal culture conditions at 2 h (Figures 6(a), 6(b), and 6(d)). 15 *μ*M zinc supplementation completely rescued the phagocytosis capacity of the RPE (Figures 6(c) and 6(d)). Phagocytosis assay at 24 h showed similar results (data not shown). Our data, therefore suggests that zinc is critically involved in RPE phagocytosis and zinc deficiency impairs this function.

### 3.6. Zinc Depletion Causes Lysosome Rupture Which Is Prevented by Zn Supplementation

**Figure 7:**
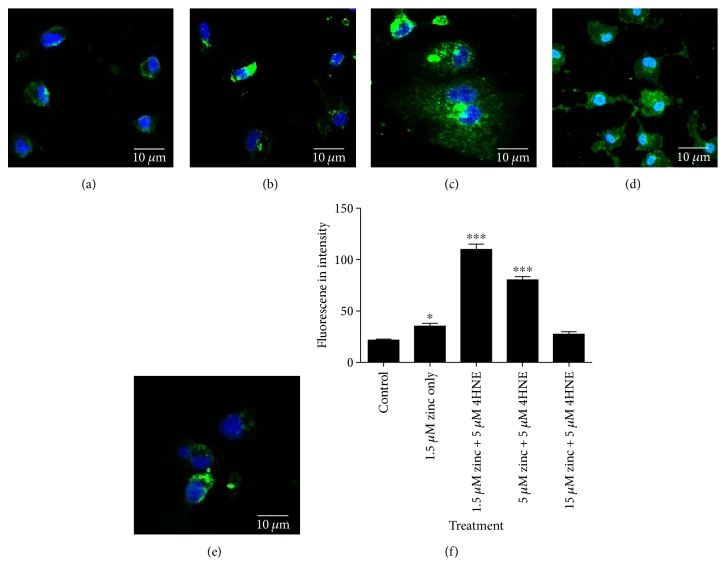
Cathepsin B staining indicating lysosomal rupture in zinc-depleted and oxidative-stressed RPE cells. RPE cells were treated with 5 M 4-HNE at 1.5 *μ*M, 5 *μ*M, and 15 *μ*M zinc concentrations as well as an untreated control and 1.5 *μ*M zinc only for 24 h before fixing the cells and immunostaining for cathepsin B. (a) Control RPE cells. (b) 1.5 *μ*M zinc only. (c) 1.5 *μ*M zinc + 4-HNE-treated RPE cells. (d) 5 *μ*M zinc + 4-HNE-treated RPE cells. (e) 15 *μ*M zinc + 4-HNE-treated RPE cells. Cell nuclei are stained with DAPI (blue) and cathepsin B (green). (f) Histogram showing the fluorescence intensity of cathepsin B staining in the different treatment groups, ^∗^*P* < 0.05 and ^∗∗∗^*P* < 0.001, compared to the control. One-way ANOVA followed by Dunnett's multiple comparison test. Fluorescence intensity of 30 cells per well was measured with three treatment replicate wells for each treatment.

To better understand the mechanism by which zinc deficiency mediates RPE cell dysfunction, we investigated RPE lysosome integrity. Loss of lysosomal integrity is implicated in impaired phagocytosis, autophagy, and various forms of cell death including apoptosis [28]. Cathepsins are major enzymes enclosed in lysosomes and help to maintain cell homeostasis by contributing to the degradation of heterophagic and autophagic material [29]. Lysosomal rupture and cathepsin B release to the cytosol are a characteristic of apoptosis induced by oxidative insult [30]. Immunostaining for cathepsin B in cells with lysosomal rupture is known to exhibit stronger and more diffuse cytoplasmic staining when compared to healthy cells with intact lysosomal membranes. Figures 7(a), 7(b), and 7(f) show that, under serum deprivation (1.5 *μ*M zinc) only, there is an increase in cathepsin B immunoreactivity compared to control (DMEM/F12+ 10% FCS), and this was further enhanced when cells were treated with 5 *μ*M 4-HNE (Figure 7(c)). Zinc supplementation dose dependently reduced cathepsin B expression in serum-free +4-HNE-treated cells (Figures 7(d) and 7(e)). These findings suggest that serum deprivation and oxidative stress induce lysosomal rupture and supplementation with zinc and can maintain RPE lysosomal integrity under oxidative stress conditions.

### 3.7. Zinc Depletion Reduces Beclin-1 Expression in ARPE19 Cells

**Figure 8:**
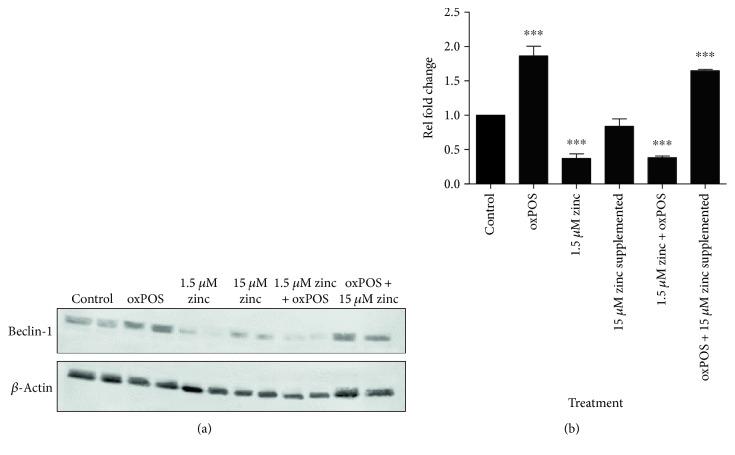
Expression of autophagy marker Beclin-1 following zinc supplementation.(a) Representative Western blot from untreated control, oxPOS treated, 1.5 *μ*M zinc, 15 *μ*M zinc, 1.5 *μ*M zinc + oxPOS treated, and oxPOS treated + 15 *μ*M zinc-supplemented RPE cells. Bands were detected for Beclin-1 at 55 KDa and *β*-actin at 40 KDa. (b) Quantification of Beclin-1 protein expression by RPE cells in the different treatment groups. Signals from Western blots were visualised by Odyssey infrared imaging system and quantified using ImageJ analysis software (version 1.45). Signals for control were set to one and the data are represented as relative fold change, ^∗∗∗^*P* < 0.001, compared to the untreated control group. One-way ANOVA followed by Dunnett's multiple comparison test. *N* = 3.

Lysosome is essential for the formation of autophagolysosome. To understand the role of zinc depletion in RPE autophagy, we measured the expression of Beclin-1 in RPE cells cultured under different conditions using Western blot. Under normal culture conditions, the addition of oxPOS increased the expression of Beclin-1 (Figures 8(a) and 8(b)). Serum depletion significantly reduced Beclin-1 expression, even with oxPOS, compared to controls (Figures 8(a) and 8(b)). When supplemented with 15 *μ*M zinc to the serum-free medium, the expression of Beclin-1 returned to levels comparable to control cells. The combination of 15 *μ*M zinc and oxPOS further increased Beclin-1 expression (Figures 8(a) and 8(b)). This result suggests that zinc deficiency may lead to autophagy dysfunction in RPE during oxidative insult.

## 4. Discussion

Under normal physiological conditions, the RPE-choroid complex contains high levels of zinc, and zinc is recognized to be important in RPE functions such as antioxidation, phagocytosis, and regulating enzymes involved in the retinal cycle as well as protein kinase activity [31, 32]. Zinc deficiency has been shown to associate with progression of diseases such as cataract and AMD [33–35]. It has been demonstrated that zinc depletion may increase oxidative stress in RPE cells, probably by decreasing the activity of antioxidant enzymes such as catalase and glutathione peroxidase [36]. Tate et al. [22] showed that zinc protected cultured RPE from H_2_O_2_ and paraquat toxicity and RPE cells in 0.55 *μ*M zinc medium contained higher levels of oxidative stress markers such as thiobarbituric acid reactive substances (TBARS), conjugated dienes, and protein carbonyls. In this study, we show that serum deprivation depleted the intracellular zinc in RPE cells, which sensitized RPE to oxidative damage and impaired RPE cell phagocytosis. We further show that zinc supplementation concentration dependently rescued oxidative stress-induced RPE damage and recovered RPE phagocytosis.

Serum contains not only zinc but also many other growth factors essential for cell survival. The increased sensitivity to oxidative damage and reduced phagocytosis of RPE cells under serum-free conditions could be caused by multiple mechanisms. However, we show that supplementation of zinc alone is sufficient to rescue RPE cells from oxidative damage during serum starvation. Our observations are in agreement with a previous study by Hyun et al. [37] where it was shown that depletion of intracellular zinc with TPEN resulted in apoptosis of cultured human RPE cells.

Exactly how zinc protects RPE cells from oxidative damage under serum-free conditions remains unknown. We found that zinc supplementation improves mitochondrial morphology and increases ATP production. Mitochondria are the main source of intracellular ROS under oxidative conditions [38]. Under serum-free and oxidative (oxPOS or 4-HNE) conditions, RPE cells expressed higher levels of intracellular ROS (e.g., higher levels of CellROX staining) and presented fragmented mitochondrial morphology and reduced ATP production. The results suggest that mitochondrial damage is critically involved in oxidative stress-induced RPE death and dysfunction under serum-free conditions. Interestingly, the addition of zinc (15 *μ*M) reduced ROS production and improved mitochondrial morphology and ATP production, suggesting that zinc can protect mitochondria from oxidative damage. A previous study has shown that zinc has a protective effect against tubular cell apoptosis following ATP depletion *in vitro* [39]. How zinc may protect mitochondria from oxidative damage and the role of zinc in mitochondrial function, biogenesis, and fusion/fission warrant further investigation.

The lysosome is the waste processing and waste disposal machinery of a cell. It not only digests materials phagocytized from exogenous sources (e.g., the formation of phagolysosome) but also plays an important role in the removal of damaged intracellular molecules and organelles (e.g., the formation of autophagosome of the autophagy pathways) [40]. In RPE cells, the lysosome is critically involved in the processing and recycling of phagocytized POS, and dysfunction of lysosomal activity may be related to the development of lipofuscin [41]. The integrity of the lysosome is essential to its function. It has been shown that lysosomal membrane destabilization may lead to the release of cathepsins into the cytosol, which may initiate the lysosomal pathway of apoptosis [42]. In addition, the released cathepsins can also amplify apoptotic signaling [43]. In the current study, we found that when RPE cells were subjected to serum-free conditions (1.5 *μ*M zinc) and the release of cathepsin B in the cytosol was significantly increased. Additional oxidative insult (5 *μ*M 4-HNE) further promoted cathepsin B release, indicative of lysosome membrane destabilization. Interestingly, zinc dose dependently prevented serum deprivation +4-HNE-induced cathepsin B release in RPE cells. Although the mechanisms related to lysosome membrane destabilization in our model system are likely to be complex and are currently unknown, our results suggest that zinc can stabilize the lysosomal membrane and maintain function.

Impaired phagocytosis of POS by RPE cells was observed in our culture system (i.e., serum deprivation). The link between zinc deficiency and decreased RPE phagocytic and lysosomal function has been suggested although not well-characterized [19]. Increased lipofuscin accumulation has been observed in zinc-deficient rats, and this phenomenon is believed to be related to elevated oxidative stress and incomplete digestion of photoreceptor outer segments in the lysosomes of the RPE [44]. The reduced phagocytosis of oxPOS by RPE cells under serum deprivation conditions may be related, at least partially, to low-zinc-mediated lysosome dysfunction.

Zinc deprivation also impaired autophagy in RPE cells evidenced by reduced Beclin-1 expression. Autophagy involves the seizing of cellular components in double-membrane vesicles known as autophagosomes and consequent delivery to lysosomes for degradation [41]. In some conditions, autophagy is considered as a physiologic prosurvival mechanism and dysregulated autophagy in RPE is associated with increased susceptibility to oxidative stress and AMD [45].

Based on the results from this study and the existing literature, it is apparent that zinc plays a key role in the regulation of apoptosis; however, the role of zinc in RPE cell autophagy remains unclear. In a recent review, Liuzzi and Cousins [46] discussed the ability of excess zinc to potentiate autophagy induced by tamoxifen, alcohol, H_2_O_2_, and dopamine as well as the suppressive effect of zinc depletion on early and late autophagy. In this study, a model with moderate zinc supplementation was used to determine the role of zinc in autophagy. There was a decreased expression of autophagy marker Beclin-1 at 1.5 *μ*M conditions, was restored when supplemented with 15 *μ*M zinc, and was significantly increased when RPE was exposed to oxPOS. This indicates that zinc depletion may lead to impaired autophagy function of RPE which could be detrimental for RPE health, but supplementation of zinc at nontoxic concentrations could be beneficial.

## 5. Conclusions

Overall, the data presented in this paper suggests that RPE cells depend upon zinc to protect against the constant insults associated with POS uptake and to maintain normal cellular function. Zinc deficiency may lead to malfunction of many intracellular organelles such as mitochondria and lysosomes, which may contribute to reduced function and increased death of RPE cells under stress conditions. The decreases in available zinc in ageing RPE and AMD could be a factor associated with decreased clearance of POS by RPE and cell death *in vivo*. Further understanding the molecular mechanisms related to zinc metabolism and homeostasis in RPE cells under aging and other pathophysiological conditions may shed light on the pathogenesis and provide crucial information on the management of various age-related retinal diseases.

## Figures and Tables

**Table 1:** Dye-conjugated probes for intracellular organelle or molecule staining.

Dye-conjugated molecular probes used in this study			
LysoTracker	1 : 100	Thermo Fisher
MitoTracker Red	1 : 100	Thermo Fisher
FluoZin-3 AM	5 *μ*M	Life Technologies
CellROX® green	5 *μ*M	Life Technologies

## References

[1] Kambe T., Tsuji T., Hashimoto A., Itsumura N. (2015). The physiological, biochemical, and molecular roles of zinc transporters in zinc homeostasis and metabolism. *Physiology Review*.

[2] Andreini C., Bertini I., Cavallaro G. (2015). Minimal functional sites allow a classification of zinc sites in proteins. *PLoS One*.

[3] Müller A., Kleinau G., Piechowski C. L. (2013). G-protein coupled receptor 83 (GPR83) signaling determined by constitutive and zinc(II)-induced activity. *PLoS One*.

[4] Csermely P., Szamel M., Resch K., Somogyi J. (1988). Zinc can increase the activity of protein kinase C and contributes to its binding to plasma membranes in T lymphocytes. *Journal of Biological Chemistry*.

[5] Ugarte M., Osborne N. N. (2014). Recent advances in the understanding of the role of zinc in ocular tissues. *Metallomics*.

[6] Ugarte M., Osborne N. N. (2001). Zinc in the retina. *Progress in Neurobiology*.

[7] Ugarte M., Grime G. W., Lord G. (2012). Concentration of various trace elements in the rat retina and their distribution in different structures. *Metallomics*.

[8] Huang L., Yu Y. Y., Kirschke C. P., Gertz E. R., Lloyd K. K. (2007). *Znt7* (*Slc30a7*)-deficient mice display reduced body zinc status and body fat accumulation. *The Journal of Biological Chemistry*.

[9] Murakami K., Kondo T., Kawase M. (1998). Mitochondrial susceptibility to oxidative stress exacerbates cerebral infarction that follows permanent focal cerebral ischemia in mutant mice with manganese superoxide dismutase deficiency. *Journal of Neuroscience*.

[10] Schraermeyer U., Peters S., Thumann G., Kociok N., Heimann K. (1999). Melanin granules of retinal pigment epithelium are connected with the lysosomal degradation pathway. *Experimental Eye Research*.

[11] Miceli M. V., Tate D. J., Alcock N. W., Newsome D. A. (1999). Zinc deficiency and oxidative stress in the retina of pigmented rats. *Investigative Ophthalmology & Visual Science*.

[12] Sekler I., Sensi S. L., Hershfinkel M., Silverman W. F. (2007). Mechanism and regulation of cellular zinc transport. *Molecular Medicine*.

[13] McCord M. C., Aizenman E. (2014). The role of intracellular zinc release in aging, oxidative stress, and Alzheimer’s disease. *Frontiers in Aging Neuroscience*.

[14] Osredkar J., Sustar N. (2011). Copper and zinc, biological role and significance of copper/zinc imbalance. *Journal of Clinical Toxicology*.

[15] Bonilha V. L. (2008). Age and disease-related structural changes in the retinal pigment epithelium. *Clinical Ophthalmology*.

[16] Julien S., Biesemeier A., Kokkinou D., Eibl O., Schraermeyer U. (2011). Zinc deficiency leads to lipofuscin accumulation in the retinal pigment epithelium of pigmented rats. *PLoS One*.

[17] Erie J. C., Good J. A., Butz J. A., Pulido J. S. (2008). Reduced zinc and copper in the retinal pigment epithelium and choroid in age-related macular degeneration. *American Journal of Ophthalmology*.

[18] Smailhodzic D., van Asten F., Blom A. M. (2014). Zinc supplementation inhibits complement activation in age-related macular degeneration. *PLoS One*.

[19] Tokarz P., Kaarniranta K., Blasiak J. (2013). Role of antioxidant enzymes and small molecular weight antioxidants in the pathogenesis of age-related macular degeneration (AMD). *Biogerontology*.

[20] Nan R., Gor J., Lengyel I., Perkins S. J. (2008). Uncontrolled zinc- and copper-induced oligomerisation of the human complement regulator factor H and its possible implications for function and disease. *Journal of Molecular Biology*.

[21] Wood J. P., Osborne N. N. (2003). Zinc and energy requirements in induction of oxidative stress to retinal pigmented epithelial cells. *Neurochemical Research*.

[22] Tate D. J., Miceli M. V., Newsome D. A. (1999). Zinc protects against oxidative damage in cultured human retinal pigment epithelial cells. *Free Radical Biology & Medicine*.

[23] Clemons T. E., Milton R. C., Klein R., Seddon J. M., Ferris F. L., Age-Related Eye Disease Study Research Group (2005). Risk factors for the incidence of advanced age-related macular degeneration in the age-related eye disease study (AREDS): AREDS report no. 19. *Ophthalmology*.

[24] Girijashanker K., He L., Soleimani M. (2008). *Slc39a14* gene encodes ZIP_14_, a metal/biocarbonate symporter: similarities to the ZIP8 transporter. *Molecular Pharmacology*.

[25] Chen M., Rajapakse D., Fraczek M., Luo C., Forester J. V., Xu H. (2016). Retinal pigment epithelial cell multinucleation in the ageing eye – a mechanism to repair damage and maintain homoeostasis. *Aging Cell*.

[26] Chen M., Forrester J. V., Xu H. (2007). Synthesis of complement factor H by retinal pigment epithelial cells is down-regulated by oxidized photoreceptor outer segments. *Experimental Eye Research*.

[27] Cho Y. E., Lomeda R. A., Ryu S. H., Lee J. H., Beattie J. H., Kwun I. S. (2007). Cellular Zn depletion by metal ion chelators (TPEN, DTPA and chelex resin) and its application to osteoblastic MC3T3-E1 cells. *Nutrition Research and Practice*.

[28] Youle R. J., van der Bliek A. M. (2012). Mitochondrial fission, fusion, and stress. *Science*.

[29] Paquet C., Sane A. T., Beauchemin M., Bertrand R. (2005). Caspase- and mitochondrial dysfunction-dependent mechanisms of lysosomal leakage and cathepsin B activation in DNA damage-induced apoptosis. *Leukemia*.

[30] Wu H., Niu H., Wu C. (2016). The autophagy-lysosomal system in subarachnoid haemorrhage. *Journal of Cellular and Molecular Medicine*.

[31] Repnik U., Stoka V., Turk V., Turk B. (2012). Lysosomes and lysosomal cathepsins in cell death. *Biochimica et Biophysica Acta (BBA) - Proteins and Proteomics*.

[32] Gleim S., Stojanovic A., Arehart E., Byington D., Hwa J. (2009). Conserved rhodopsin intradiscal structural motifs mediate stabilization: effects of zinc. *Biochemistry*.

[33] Rezaei K. A., Chen Y., Cai J., Sternberg P. (2008). Modulation of Nrf2-dependent antioxidant functions in the RPE by Zip2, a zinc transporter protein. *Investigative Ophthalmology & Visual Science*.

[34] Newsome D. A., Miceli M. V., Tate D., Alcock N. W., Oliver P. (1996). Zinc content of human retinal pigment epithelium decreases with age and macular degeneration, but superoxide dismutase activity increases. *The Journal of Trace Elements in Experimental Medicine*.

[35] Richardson N. L., Higgs D. A., Beames R. M., Mcbride J. R. (1985). Influence of dietary calcium, phosphorus, zinc and sodium phytate level on cataract incidence, growth and histopathology in juvenile chinook salmon (*Oncorhynchus tshawytscha*). *The Journal of Nutrition*.

[36] Grahn B. H., Paterson P. G., Gottschall-Pass K. T., Zhang Z. (2001). Zinc and the eye. *Journal of the American College of Nutrition*.

[37] Hyun H. J., Sohn J. H., Ha D. W., Ahn Y. H., Koh J. Y., Yoon Y. H. (2001). Depletion of intracellular zinc and copper with TPEN results in apoptosis of cultured human retinal pigment epithelial cells. *Retinal Cell Biology*.

[38] Starkov A. A. (2008). The role of mitochondria in reactive oxygen species metabolism and signaling. *Annals of the New York Academy of Sciences*.

[39] Wei Q., Wang J., Wang M. H., Yu F., Dong Z. (2004). Inhibition of apoptosis by Zn^2+^ in renal tubular cells following ATP depletion. *American Journal of Physiology - Renal Physiology*.

[40] Glick D., Barth S., Macleod K. F. (2010). Autophagy: cellular and molecular mechanisms. *The Journal of Pathology*.

[41] Guha S., Liu J., Baltazar G., Laties A. M., Mitchell C. H. (2014). Rescue of compromised lysosomes enhances degradation of photoreceptor outer segments and reduces lipofuscin-like autofluorescence in retinal pigmented epithelial cells. *Advances in Experimental Medicine and Biology*.

[42] Johansson A. C., Appelqvist H., Nilsson C., Kågedal K., Roberg K., Ollinger K. (2010). Regulation of apoptosis-associated lysosomal membrane permeabilization. *Apoptosis*.

[43] Turk B., Stoka V. (2007). Protease signalling in cell death: caspases versus cysteine cathepsins. *FEBS Letters*.

[44] Kennedy C. J., Rakoczy P. E., Robertson T. A., Papadimitriou J. M., Constable I. J. (1994). Kinetic studies on phagocytosis and lysosomal digestion of rod outer segments by human retinal pigment epithelial cells *in vitro*. *Experimental Cell Research*.

[45] Mitter S. K., Song C., Qi X. (2014). Dysregulated autophagy in the RPE is associated with increased susceptibility to oxidative stress and AMD. *Autophagy*.

[46] Liuzzi J. P., Cousins R. J. (2014). Mammalian zinc transporters. *Annual Review of Nutrition*.

